# miRNA-451a and miRNA-125a Expression Levels in Ankylosing Spondylitis: Impact on Disease Diagnosis, Prognosis, and Outcomes

**DOI:** 10.1155/2020/2180913

**Published:** 2020-12-28

**Authors:** Dina Salem Fotoh, Rasha Ibrahim Noreldin, Mohamed Soliman Rizk, Maha Mohamed Elsabaawy, Heba Ahmed Esaily

**Affiliations:** ^1^Physical Medicine, Rheumatology and Rehabilitation Department, Faculty of Medicine, Menoufia University, Egypt; ^2^Clinical Pathology Department, Faculty of Medicine, Menoufia University, Egypt; ^3^Medical Biochemistry and Molecular Biology Department, Faculty of Medicine, Menoufia University, Egypt; ^4^Hepatology and Gastroenterology Department, National Liver Institute, Menoufia University, Egypt

## Abstract

**Background:**

Early diagnosis of ankylosing spondylitis (AS) is yet not elucidated, with a potential diagnostic glance of microRNAs (miRNAs).

**Aim:**

Study the expression profile of miRNA-451a and miRNA-125a in AS patients and their impact on disease activity and prognosis.

**Methods:**

A cross-sectional study included 55 AS patients diagnosed according to modified New York criteria in 1984 with 55 matched healthy controls. History, clinical examination, and disease activity assessment with Bath ankylosing spondylitis disease activity index (BASDAI) were done. Full laboratory and radiological assessment along with expression profile of m iRNA-451a and miRNA-125a were tabulated and analyzed.

**Results:**

Higher expression levels of miRNA-125a and lower of miRNA-451a in AS patients compared to controls. Furthermore, miRNA-125a expression was high in active AS patients compared to inactive patients and controls (7.0 ± 3.4 vs. 4.1 ± 2.1 vs. 2.6 ± 0.6, *p* < 0.001, respectively). miRNA-451a was significantly lower in active AS patients compared to inactive patients and controls (2.2 ± 1.1 vs. 4.1 ± 2.3 vs. 7.1 ± 4.5, respectively). Both miRNAs (miRNA-125a and miRNA-451a) had evident accuracy for AS diagnosis with areas under the curve (AUC) of 0.788 and 0.802, respectively. miRNA-125a had potential impact on AS activity with AUC of 0.777. Plasma levels of both miRNAs were able to distinguish AS patients with structural damage with AUCs 0.775 and 0.692, respectively.

**Conclusions:**

Both miRNA-451a and miRNA-125a were found to be of great value as sensitive noninvasive diagnostic, prognostic, and disease burden biomarker of AS patients in Egypt with suggested further studies for future therapeutic implications.

## 1. Introduction

Ankylosing spondylitis (AS) is an autoimmune inflammatory disease of young adults affecting the axial skeleton and articular joints, particularly the sacroiliac joints. The inflammation also affects tendons, entheseal structures, peripheral joints, and cartilaginous tissue lasting for years before irreversible damage occurs [1] in addition to extra-articular manifestations including uveitis, psoriasis, and inflammatory bowel disease [2].

However, as the exact pathogenesis is still unclear, it is thought that the pathological process of AS starts with an early inflammatory phase followed by new bone formation inducing local osteitis, cartilage erosion, bone destruction, and subsequent ankylosis. Sacroiliitis and syndesmophytes (radiographic AS) are detected by plain radiography, while earlier detection could be achieved by magnetic resonance imaging (MRI) [3].

Human leukocyte antigen (HLAB-27) is still the best biomarker for AS diagnosis, and C-reactive protein (CRP) is the best marker for disease activity assessment, determining treatment efficacy and structural progression [4]. However, HLAB-27 is responsible for about only 30% of the genetic factors for AS, indicating that there are other genetic disorders involved in AS pathogenesis [5].

MicroRNAs (miRNAs) that function as regulators of posttranscriptional control of gene expression are small, noncoding RNAs 20 to 25 nucleotides in length. While most of miRNAs are intracellular, a cell-free form significant number is present in the circulation. These extracellular miRNAs have remarkable stability under extreme conditions like extended storage. Dysregulation of miRNA expression and target genesis related to the pathophysiology of many disorders such as AS, cancer, and autoimmune rheumatic diseases making these circulating miRNAs have valuable diagnostic and prognostic roles in several diseases [6, 7].

Recently, the important role of circulating miRNAs in AS pathogenesis has been explored [8]. T cells and peripheral blood mononuclear cells (PBMCs) of AS patients have shown an altered miRNA expression which is involved in specific pathways in the disease pathogenesis [8, 9]. Low expression of miRNA-451a and miR-150-5p with high expression of miRNA46a-5p, miRNA-125a, miR-151a-3p, and miR-22-3p was observed in PBMCs of AS patients [10].

## 2. Aim of the Work

Firstly, study the expression profile of circulating miRNA-451a and miRNA-125a in AS patients and if they are associated with the disease itself. Secondly, evaluate the role of these specific miRNAs as potential biomarkers reflecting disease activity and structural damage.

## 3. Patients and Methods

The sample sizing assumes that the expected specificity for circulating microRNAs in AS patients (prevalence 1-1.4%) is 92.5% [10]. To achieve 80% power to detect this difference using an equation based on specificity = *Z*2 − *α*^2^ × SP × (1 − SP)*L*^2^ × (1 − prevalence), with a significance level 5%, it is estimated that 110 participants would be required.

This cross-sectional study was conducted on 55 AS patients recruited from the outpatient rheumatology clinic of our university between November 2018 and November 2019 diagnosed according to the modified New York criteria 1984 AS [11] with 55 healthy controls of matched age and gender. This study was approved with the research ethical committee of our university (IRB number: 191018INTPH43). An informed consent was obtained from all subjects participating in this study. This study excluded patients with chronic infections, pregnancy, malignancies, other rheumatologic diseases and patients on biologics.

Demographic data were recorded for all subjects. For AS patients, disease duration, special habits, especially smoking, clinical assessment, and history of administered therapy were taken. Quality of life was assessed using the AS quality-of-life-questionnaire (ASQoL) [12]. Pain was recorded on a 10 cm visual analog scale (VAS) [13].

Bath AS disease activity index (BASDAI) with C-reactive protein (CRP) (mg/l) and erythrocyte sedimentation rate (ESR) (mm/h) was used to assess disease activity [14]. Mobility and functional limitations were assessed by Bath AS metrology index (BASMI) [15] and Bath AS functional index (BASFI) [16].

Patients were classified into two groups based on CRP and ESR and BASDAI: active AS patients with a CRP level > 8 mg/l and/or with a BASDAI score ≥ 4 and ESR > 20 mm/h, while other patients were defined as inactiv AS cases.

Full laboratory investigations including genetic factors (HLA-B27), antigen, inflammatory markers (ESR and CRP) by the Westergren method, and 25-hydroxyvitamin D [25(OH) D] levels by enzyme immunoassay method using a homogenous enzyme-coupled to vitamin D binding protein were done.

Lateral radiographs of the lumbar and cervical spines were done, and structural damage was assessed according to the presence or absence of syndesmophytes by modified stroke ankylosing spondylitis spinal score (MSASSS) from the anterior margins of lower border of C2 to the upper border of Th1 and from the lower border of Th12 to the upper border of S1, graded from 0 to 3 points each (0: normal; 1: erosion, sclerosis, or squaring; 2: syndesmophyte; 3: bone bridge) with a total score of 0-72 [17].

### 3.1. Samples and RNA Isolation

The whole blood samples were collected to EDTA tubes, and within 4 hours, miRNA was extracted from the samples using miRNeasy®, Mini Kit (50), Qiagen, Germany. miRNA extracted by kit was stored at −80. When both patients and control samples were collected, the counter miRNA array was performed before the complementary DNA (cDNA). cDNA synthesis for all miRNAs by using primers of Miscript® II RT kit, Qiagen, Germany, was done [9].

### 3.2. miRNA Analysis

Quantitative analysis was done by using real-time RT-PCR validation 7500 Applied Biosystem, Germany, using Miscript SYBR green, Qiagen, and specific miRNA primer assay for miRNA-125a-5p and miRNA-451a and housing keeping primer assay (SNORD 68) as an internal control. Relative miRNA expression level for each sample was calculated by the equation: the relative miRNA expression = (2 − ΔCt) × 100.

## 4. Statistical Analysis

The data were analyzed using the IBM SPSS software package version 20. Comparisons between groups for categorical variables were assessed using chi-square test. Student *t*-test was used to compare two groups for normally distributed quantitative variables while ANOVA test was used for comparing the three studied groups followed by post hoc test for pairwise comparison. Kruskal-Wallis test was used to compare different groups for abnormally distributed quantitative variables followed by post hoc tests for pairwise comparison. Mann-Whitney test was used to compare two groups for abnormally distributed quantitative variables. Spearman coefficient was used to correlate between abnormally quantitative variables, while Pearson coefficient was used to correlate between normally quantitative variables. Receiver operating characteristic curve (ROC) was used to determine the diagnostic performance of the markers in terms of sensitivity and specificity. For all statistics, a *p* value of ≤0.05 was statistically significant and ≤0.001 was highly significant [18].

## 5. Results

Fifty-five AS patients classified into active and inactive groups based on CRP and ESR and BASDAI with equal number of age- and gender-matched controls were included.

The clinical features of AS patients (*n* = 55) and the healthy controls (*n* = 55) were included in Table 1. Active AS patients included 20 males (69%) and 9 females (31%) with mean age of 35.7 ± 7.9 years and disease duration of 25 ± 2.4 years. Inactive AS patients included 19 males (73.1%) and 7 females (26.9%) with mean age of 37 ± 10.2 years and disease duration of 22 ± 5.1 years. Controls were 33 males (60%) and 22 females (40%) with a mean age of 40.3 ± 10 years. 36 (65.4%) AS patients showed HLAB-27 antigen (Table 1).

There were no significant differences regarding age, sex, and occupation between patients and controls. Regarding disease duration, there were significant differences between both groups of AS patients (*p* = 0.005). Significant differences regarding BASDAI, BASFI, BASMI, MSASSS, and ASQoL between active and inactive AS patients were found. Inflammatory markers (ESR and CRP) were significantly higher in AS patients than in healthy controls. There were significant differences regarding VAS and vitamin D between patients and controls (*p* < 0.001) (Table 1).

**Figure 1:**
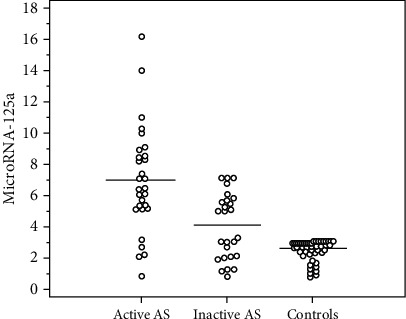
Comparison between the three studied groups according to microRNA-125a.

**Figure 2:**
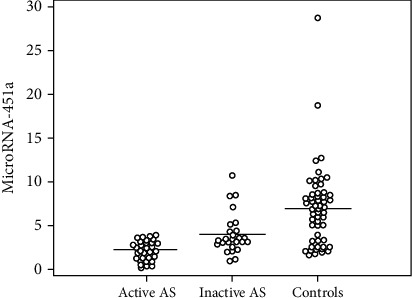
Comparison between the three studied groups according to microRNA-451a.

To investigate the potential usefulness of both types of miRNAs as disease activity biomarkers, we analyzed the association of these plasma miRNAs with the activity of AS. miRNA-125a was significantly high in active AS patients compared to inactive patients and controls (7.0 ± 3.4 vs. 4.1 ± 2.1 vs. 2.6 ± 0.6), respectively. miRNA-451a was significantly lower in active AS patients compared to inactive patients and controls (2.2 ± 1.1 vs. 4.1 ± 2.3 vs. 7.1 ± 4.5), respectively (Table 1 and Figures 1 and 2).

In AS patients, miRNA-125a was positively correlated with CRP, ESR, BASDAI, BASFI, and BASMI (*p* < 0.001) with negative correlation with vitamin D (*r* = −0.641 and *p* < 0.001). This means that active AS patients with higher miRNA-125a have higher inflammatory markers with higher disease activity scores but have lower levels of vitamin D compared to inactive patients. miRNA-451a was negatively correlated with CRP, ESR, BASDAI, BASFI, and BASMI (*p* < 0.001) with positive correlation with vitamin D (*r* = 0.471 and *p* < 0.001). There was a nonsignificant correlation between both types of miRNAs (miRNA-125a and miRNA-451a) and disease duration, indicating that disease duration had no effect on circulating miRNA levels (*p* = 0.879 and *p* = 0.884, respectively) (Table 2).

To investigate the accuracy of both miRNA-125a and miRNA-451a as potential diagnostic biomarkers discriminating AS patients from healthy controls, ROC curve analysis was performed. Both miRNA-125a and miRNA-451a had an area under the curve (AUC) of 0.788 and 0.802, respectively, and the optimal cutoff points of them were 3.029 and 3.372, respectively. miRNA-125a had sensitivity and specificity of 70.91% and 83.64%, respectively. miRNA-451a had sensitivity and specificity of 65.45% and 78.18%, respectively (Table 3).

To estimate that both plasma miRNAs can be used as potential biomarkers for assessment of AS disease activity, ROC curve analyses were performed. miRNA-125a had an AUC of 0.777, and the optimal cutoff point was 5.815 with sensitivity and specificity of 62.07% and 80.77%, respectively, while miRNA-451a had an AUC of 0.782, and the optimal cutoff point was 2.979 with sensitivity and specificity of 68.97% and 76.925, respectively (Table 3).

Regarding structural damage, AS patients were separated into two subgroups measured by presence (*n* = 32) or absence (*n* = 23) of syndesmophytes. The plasma levels of these miRNAs could accurately distinguish AS patients with radiographic severity. miRNA-125a had an AUC of 0.775, and the optimal cutoff point was 5.637 with sensitivity and specificity of 65.62% and 78.265, respectively. miRNA-451a had an AUC of 0.692, and the optimal cutoff point was 2.979 with sensitivity and specificity of 62.50% and 73.915%, respectively (Table 3).

Upregulation of miRNA-125a along with downregulation of miRNA-451a together with the disease duration was the only predictor of AS disease activity by univariate logistic regression (*p* = 0.002, *p* = 0.003, and *p* = 0.013, respectively) (Table 4).

## 6. Discussion

AS is a chronic autoimmune inflammatory disorder characterized by pathologic new bone formation [19]. The etiology, pathogenesis, and diagnosis of AS are still challenging. Due to its overwhelming prevalence in AS, HLA-B27 antigen has been used as an important diagnostic biomarker. However, it accounts for less than 30% of the overall genetic risks of AS (6). Moreover, X-ray cannot detect early radiological changes, and MRI cannot be used as a screening test due to cost and unfavorable manipulation. Therefore, novel biomarkers are urgently needed for AS screening and diagnosis.

The precise diagnostic and prognostic concerns of AS are still unmet medical needs. Care of AS cases should be prioritized for the destructive nature of this chronic inflammatory disorder affecting young adults and endangering their activities, productivity, and quality of life, with consequent national economic burden [19, 20].

Genetic polymorphisms of miRNAs and their targets might alter the risk of AS development. In this study, both miRNA-125a and miRNA-451a were evaluated as potential diagnostic and prognostic markers AS reflecting disease activity and structural damage in 55 cohorts in our country.

Active AS patients were younger than inactive cases (35.7 ± 7.9 years) with longer disease duration (25 ± 2.4 years) and were mostly males (69%), which is consistent with the usual pattern of the disease in which males are affected 2 to 4 times more frequently than females. Our results correspond with the published data in literature reflecting the youthful nature of the disease being more aggressive in young adults with peak age of onset between second and third decades [19]. Most studies had incriminated male sex and longer disease duration as poor prognostic indices of AS outcome [21]. However, functional incapacities and structural damage were reported in both genders [22].

In the current study, vitamin D was deficient and insufficient in active and inactive groups of AS, respectively, with significant more reduction in active AS cases, which might reflect the potential substantial role of deficient vitamin D levels in disease pathogenesis and activity. This comes in agreement with several recent studies which had substantiated the role of vitamin D in AS through both adaptive and innate immune function rather than calcium metabolic effects [19, 23], implying the value of vitamin D supplementation to this special ill cohort [24].

However, there are contradictory reviews about the association between vitamin D and AS that had been published like the study of Zhao et al., who denied any immunomodulatory role of vitamin D in AS disease activity [25].

HLAB-27 has been used to be the mainstay of AS diagnosis despite the well-known presence of the class 1 human leucocyte antigen HLA-B27 grants only 30% of the total genetic risk to AS [26]. In the study of Kocyigit et al., HLAB-27 was prevalent in 96.8% in a Caucasian cohort [23]. In this study, HLAB-27 was prevalent in 65.4% of AS cases. This discrepancy might be yielded to the racial genetic variations of studied populations. The results might authenticate the search of new AS diagnostic markers with more advantageous accuracy.

At molecular level, miRNA alterations had been considered as sensitive potential markers of many pathologic conditions. In AS, immunopathogenesis and bone remodeling are the mainstays involved in disease pathogenesis. Accordingly, miRNAs might be implicated in AS diagnostic, prognostic, and therapeutic processes [8, 10].

In this current study, there was an alteration in the expression profile of both miRNA-125a and miRNA-451a levels between AS patients and controls, indicating that the altered levels of these miRNAs could accurately diagnose AS patients. Analysis of the ROC curve to prove the diagnostic potential of these two miRNAs showed an evident diagnostic accuracy (AUC ranged from 0.788 to 0.802) making them the suitable biomarkers for AS diagnosis. Regarding AS disease activity, miRNA-125a was proved to be significantly higher and miRNA-451a was significantly lower in active AS patients compared to inactive patients and controls, indicating that the altered levels of miRNA-125a could accurately assess AS disease activity together with the ROC curve analysis which demonstrated evident diagnostic accuracy of this miRNA for disease activity.

This comes in agreement with the study by Perez-Sanchez et al. who had studied the ROC curves of six miRNAs including miRNA-125a-5p, miR-151a-3p, miR-22-3p, miRNA-150-5p, and miRNA-451a and reported a moderate distinguishing efficiency, with the AUCs for these miRNAs ranged from 0.614 to 0.781 (*p* < 0.05) [10].

In AS, the structural damages are considered the eventual devastating destiny starting from new bone formation and syndesmophyte up to ankylosis of the sacroiliac joints and vertebral column [9, 27]. Several studies identified some miRNAs involved in bone remodeling and regulation of osteogenesis and osteoclastogenesis, via interaction with signaling molecules that control these processes [28, 29]. miRNA-125a was known to inhibit osteoblastic activity added to the adverse impact on osteoclastogenesis via TRAF6/nuclear factor of activated T cell 1 (NFATc1)/miRNA-125a regulatory feedback [29, 30]. miRNA-451a was reported to alter bone formation by means of subdued phosphorylation of p38 mitogen-activated protein-kinase (MAPK) [29, 31].

AS structural damage, which is evaluated by the presence of syndesmophytes, was distinguished by both miRNA-125a and miRNA-451a in AS patients with syndesmophyte structural damage with evident diagnostic accuracy (AUCs of 0.775 and 0.692, respectively). These results proposed an essential role of miRNAs in discriminating AS structural damage and radiographic severity.

This comes in agreement with the studies by Perez-Sanchez et al. and Mohammadi et al. Perez-Sanchez et al. reported that plasma miRNA-125a and miRNA-451a along with other two miRNAs were found to be appropriate biomarkers for the prediction of AS structural damage with marked diagnostic accuracy, which is evidenced by an AUC of 0.820 (*p* < 0.002), at a sensitivity of 82.4% and a specificity of 80.0% from a cutoff value of 0.633 [10, 32].

The association between miRNAs and inflammatory markers reflecting disease activity was tested through transfecting lymphocytes of AS cases with three selected miRNA mimics (miRNA-125a, miRNA-451a, and miRNA-22-3p mimics). A momentous decline in those inflammatory markers had glanced light on their potential roles in disease pathogenic pathways, with probable prospective therapeutic influences [33].

In the current study, the linkage between the two selected miRNAs and markers of AS disease activity (CRP, ESR, BASDAI, BASFI, and BASMI) was substantiated statistically significant through positive correlation with miRNA-125a (*p* < 0.001) and negative with miRNA-451a (*p* < 0.001), supporting the hypothesis that both miRNAs might be involved in inflammatory processes of AS.

To our utmost knowledge, this was the first study connecting both miRNA (miRNA-125a and miRNA-451a) with AS diagnosis, activity, and disease burden in AS cohorts of our country with their own genetic properties.

Additionally, the power of upregulation of miRNA-125a along with downregulation of miRNA-451a and longer disease duration was assessed and valued as the only predictor of AS activity by univariate logistic regression.

## 7. Limitations of the Study

More large-scale prospective studies are mandated to validate the diagnostic and prognostic burden of these two biological markers in AS. The suggested therapeutic influences rather than the diagnostic implications of these sensitive biomarkers should be the prospective target of upcoming research.

## 8. Conclusion

Both miRNA-451a and miRNA-125a were found to be key in AS pathogenesis and of great value as diagnostic, prognostic, and disease burden biomarkers in AS patients in our country with suggested future therapeutic implications which deserve further studies.

## Figures and Tables

**Table 1:** Comparison between the three studied groups according to different parameters.

	Active AS (*n* = 29)	Inactive AS (*n* = 26)	Controls (*n* = 55)	*p*
Age (years)				
*Mean* ± *SD*	35.7 ± 7.9	37 ± 10.2	40.3 ± 10	0.087
Sex				
Male	20 (69%)	20 (76.9%)	33 (60%)	0.304
Female	9 (31%)	6 (23.1%)	22 (40%)	
Occupation				
Working	19 (65.5%)	19 (73.1%)	38 (69.1%)	0.832
Not working	10 (34.5%)	7 (26.9%)	17 (30.9%)	
Smoking				
Smoker	13 (44.8%)	9 (34.6%)	10 (18.2%)	0.030
Non smoker	16 (55.2%)	17 (65.4%)	45 (81.8%)	
MicroRNA-125a	7.0 ± 3.4	4.1 ± 2.1	2.6 ± 0.6	<0.001
Sig.bet.Grps	*p*_1_ < 0.001, *p*_2_ < 0.001, *p*_3_ < 0.001			
MicroRNA-451a	2.2 ± 1.1	4.1 ± 2.3	7.1 ± 4.5	<0.001
Sig.bet.Grps	*p*_1_ = 0.005, *p*_2_ < 0.001, *p*_3_ = 0.007			
Diseases duration	22 ± 5.1	25 ± 2.4	–	0.005
BASDAI	5 ± 0.7	2.6 ± 0.8	–	<0.001
BASFI	4.7 ± 1.0	2.5 ± 0.7	–	<0.001
BASMI	4.6 ± 0.5	2.4 ± 0.6	–	<0.001
MSASSS	31 ± 2	17.6 ± 2.1	–	<0.001
Nonstructural damage (≤20)	(0 0%)	23 (88.5%)	–	<0.001
Structural damage (>20)	29 (100%)	3 (11.5%)	–	
ASQoL	9.9 ± 1.8	5.6 ± 2.2	–	<0.001
VAS	6.9 ± 1.3	4.1 ± 0.9	–	<0.001
ESR	34.4 ± 6.7	10.3 ± 4.7	3.8 ± 0.7	<0.001
Sig.bet.Grps	*p*_1_ < 0.001, *p*_2_ < 0.001, *p*_3_ < 0.001			
CRP	49.6 ± 8.6	6.8 ± 0.8	3.3 ± 0.9	<0.001
Sig.bet.Grps	*p*_1_ < 0.001, *p*_2_ < 0.001, *p*_3_ = 0.005			
Vitamin D	16.3 ± 2.8	23.5 ± 1.9	37.2 ± 6.6	<0.001
Sig.bet.Grps	*p*_1_ < 0.001, *p*_2_ < 0.001, *p*_3_ < 0.001			
HLAB-27	20 (69%)	16 (61.5%)	–	0.563

*n*: number; BASDAI: Bath AS disease activity index; BASFI: Bath AS functional index; BASMI: Bath AS metrology index; VAS: visual analog scale; MSASSS: modified stroke ankylosing spondylitis spinal score; ASQoL: ankylosing spondylitis quality of life questionnaire; ESR: erythrocyte sedimentation rate; CRP: C-reactive protein; *p*: *p* value for comparing between the studied groups; *p*_1_: *p* value for comparing between active AS and inactive AS; *p*_2_: *p* value for comparing between active AS and controls; *p*_3_: *p* value for comparing between inactive AS and controls. Statistically significant at *p* ≤ 0.05.

**Table 2:** Correlation between microRNA-125a and microRNA-451a with laboratory parameters in the patient group (*n* = 55).

	MicroRNA-125a		MicroRNA-451a	
	*r*	*p*	*r*_*s*_	*p*
CRP	0.584^∗^	<0.001^∗^	-0.473^∗^	<0.001^∗^
ESR	0.631^∗^	<0.001^∗^	-0.470^∗^	<0.001^∗^
Vitamin D	-0.641^∗^	<0.001^∗^	0.471^∗^	<0.001^∗^
BASDAI	0.625^∗^	<0.001^∗^	-0.470^∗^	<0.001^∗^
BASFI	0.635^∗^	<0.001^∗^	-0.467^∗^	<0.001^∗^
BASMI	0.597^∗^	<0.001^∗^	-0.480^∗^	<0.001^∗^
Diseases duration	-0.030	0.879	-0.028	0.884

*r*: Pearson coefficient; *r*_*s*_: Spearman coefficient. Statistically significant at *p* ≤ 0.05. BASDAI: Bath AS disease activity index; BASFI: Bath AS functional index; BASMI: Bath AS metrology index; ESR: erythrocyte sedimentation rate; CRP: C-reactive protein.

**Table 3:** ROC curve analysis for microRNA-125a and microRNA-451a to diagnose structural damage in AS patients.

	AUC	*p*	Cutoff	Sensitivity (CI = 95%)	Specificity	PPV	NPV	Accuracy
To distinguish AS patients from controls								
MicroRNA-125a	0.788	<0.001	3.029	70.91	83.64	81.2	74.2	77.3
MicroRNA-451a	0.802	<0.001	3.372	65.45	78.18	75.0	69.4	71.8
To diagnose active AS patients from inactive patients								
MicroRNA-125a	0.777	<0.001	5.815	62.07	80.77	78.3	65.6	70.9
MicroRNA-451a	0.782	<0.001	2.979	68.97	76.92	76.9	69.0	72.7
To detect structural damage in AS patients								
MicroRNA-125a	0.775	0.001	5.637	65.62	78.26	80.8	62.1	70.9
MicroRNA-451a	0.692	0.016	2.979	62.50	73.91	76.9	58.6	67.3

AUC: area under a curve; *p* value: probability value; CI: confidence intervals; NPV: negative predictive value; PPV: positive predictive value. Statistically significant at *p* ≤ 0.05.

**Table 4:** Univariate and multivariate analyses for the parameters affecting active AS.

	Active AS	Inactive AS	*p*	OR (95% CI)
MicroRNA-125a	7.0 ± 3.4	4.1 ± 2.1	0.298^∗^	1.195 (0.854–1.671)
MicroRNA-451a	2.2 ± 1.1	4.1 ± 2.3	0.004^∗^	0.191 (0.061–0.594)
Diseases duration	22 ± 5.1	25 ± 2.4	0.003^∗^	1.692 (1.195–2.397)

OR: odds ratio; CI: confidence interval; LL: lower limit; UL: upper limit. ^#^All variables with *p* < 0.05 were included in the multivariate. ^∗^Statistically significant at *p* ≤ 0.05.

## Data Availability

Data used to support the findings of this study are included within the article.

## Additional Points

*Significance and Innovation*. Ankylosing spondylitis is an invasive autoimmune disorder with difficult diagnostic, prognostic, and disease burden evaluation challenges. MiRNAs are very sensitive and promising biomarkers. The role both miRNA-451a and miRNA-125a was justified to be of significance as a sensitive noninvasive diagnostic, prognostic, and disease activity and burden biomarkers of AS in Egypt. Validation of both biomarkers has suggested further studies for future therapeutic implications.
